# Silencing of circTASP1 inhibits proliferation and induces apoptosis of acute myeloid leukaemia cells through modulating miR‐515‐5p/HMGA2 axis

**DOI:** 10.1111/jcmm.16765

**Published:** 2021-07-01

**Authors:** Yuanyuan Lin, Yan Huang, Changda Liang, Shupei Xie, An Xie

**Affiliations:** ^1^ Department of Hematology/Oncology Jiangxi Provincial Children's Hospital Nanchang China; ^2^ Department of Lymphatic and Hematologic Oncology Jiangxi Provincial Cancer Hospital Nanchang China; ^3^ Jiangxi Institute of Urology The First Affiliated Hospital of Nanchang University Nanchang China

**Keywords:** apoptosis, CircTASP1, HMGA2, miR‐515‐5p, proliferation

## Abstract

Acute myeloid leukaemia (AML) is a common hematopoietic disease that is harmful to the lives of children and adults. CircRNAs are aberrantly expressed in the haematologic malignancy cells. However, the expression of circTASP1 and its function in AML remain unclear. In this study, we showed that circTASP1 was significantly up‐regulated in AML peripheral blood samples and cells. Knockdown of circTASP1 inhibited proliferation and promoted apoptosis of HL60 and THP‐1 cells in vitro. Bioinformatics prediction and luciferase reporter assay proved that circTASP1 sponged miR‐515‐5p and negatively regulated miR‐515‐5p expression in HL60 and THP‐1 cells. High mobility group A2 (HMGA2) was proved to be a downstream target of miR‐515‐5p. The rescue experiments confirmed that knockdown of circTASP1 inhibited proliferation and induced apoptosis by modulating miR‐515‐5p/HMGA2 pathway. Moreover, the in vivo experiment indicated that knockdown of circTASP1 suppressed tumour growth. In conclusion, circTASP1 acts as a sponge for miR‐515‐5p to regulate HMGA2, thereby promoting proliferation and inhibiting apoptosis during AML progression. Thus, circTASP1 has the potential to be explored as a therapeutic target for AML treatment.

## 1 INTRODUCTION

Acute myeloid leukaemia (AML) is a haematopoietic disease caused by aberrant self‐renewal, proliferative and potential differentiation in the precursor of bone marrow cells.^1^ AML in children is rare and heterogeneous, with an incidence of 7 cases per million children under the age of 15 years. In developed countries, intensive treatment combined with effective supportive care can improve the survival rate to 70%.^2^ Stem cell transplantation is a common treatment strategy for AML.^3^ Advances in molecular genetics have facilitated the identification of prognostic markers for AML, and the molecular abnormalities and cytogenetic results are considered to be the most vital factors. Approximately 50% of patients with AML have no cytogenetic abnormalities which are considered as the major component of intermediate‐risk AML.^4^ At present, accumulating studies have identified more prognostic markers, including a variety of molecular signals: DNA mutation, abnormal expression of miRNA and non‐coding RNA (ncRNA).^5^ These potential biomarkers will contribute to the diagnosis, monitoring or prognosis of AML.

Non‐coding RNA (ncRNA) is a class of non‐protein translation RNA, which includes long lncRNA, miRNA and circRNA. CircRNAs are characterized by covalent closed loops and are formed by back‐splicing without 3’‐end and 5’‐end.^6^ Nowadays, circRNAs are common in eukaryotes and are abundant and conserved in cytoplasm and blood.^7^ Circ‐ANAPC7 is reported to be up‐regulated in AML.^8^ In addition, circRNA‐DLEU2 expression is enhanced in AML tissues and cells, which promotes proliferation and inhibits cell apoptosis of AML cells. It accelerates human AML in vivo by reducing miR‐496 and enhancing PRKACB expression.^9^ Knockdown of circ_0009910 represses AML cell growth in vitro and in vivo via increasing miR‐20a‐5p.^10^ Considering the expression change of circRNAs in AML, the expression change of circRNAs might be involved in leukaemogenesis. However, there are still many circRNAs that need to be investigated, which may participate in the development or response to the treatment of AML.

In this study, circRNA microarray GSE116617 and circRNA sequencing are used to identify the expression of circRNAs in peripheral blood samples from 8 patients with AML and 4 normal individuals. Furthermore, circ_406083 (Gene symbol: TASP1) was identified to be significantly up‐regulated in patients with AML compared with normal, which was consistent with a previous study.^11^ In addition, high levels of circTASP1 predicted the poor prognosis of patients with AML. Further in vitro and in vivo experiments confirmed that down‐regulation of circTASP1 inhibited the proliferation of AML cells and promoted apoptosis by increasing the expression of miR‐515‐5p in AML cells, thereby providing a novel target for AML treatment.

## 2 MATERIALS AND METHODS

In this study, the animal experiments were conducted in accordance with the Guide for the Care and Use of Laboratory Animals, and the acquisition of human samples was based on the principle of Declaration of Helsinki and was authorized by the Medical Ethics Committee of The First Affiliated Hospital of Nanchang University.

### 2.1 Human samples and circRNA microarray analysis

Peripheral blood samples from 60 patients with AML and 22 normal controls were obtained following informed consent at Jiangxi Provincial Children's Hospital. All procedures in this study involving human participants were performed in accordance with the standards upheld by the Ethics Committee of Jiangxi Provincial Children's Hospital and with those of the 1964 Helsinki Declaration and its later amendments for ethical research involving human participants. Twelve samples (4 normal and 8 AML) were used for the circRNA microarray. After RNA isolation, circRNA was enriched via digesting linear RNA with RNase R (Epicentre) and then was amplified with RT‐PCR and labelled with Arraystar Human CircRNA Array. Finally, the results were scanned using Agilent Scanner G2505C. The Gene Expression Omnibus (GEO) accession number is GSE116617.

### 2.2 Cell culture and transfection

The human myeloid leukaemia cell lines (HL60, KG‐1, U937 and THP‐1) and HS‐5 cells were purchased from American Type Culture Collection (ATCC) and cultured in RPMI‐1640 medium (Gibco) containing 10% foetal bovine serum (FBS; Gibco) at 37℃ in 5% CO_2_.

The CD34+cell from peripheral blood samples were separated by centrifugation, washed and isolated using a CD34^+^ isolation kit (Miltenyi Biotec) according to the manufacturer's protocol.^12^

A total of 5 × 10^6^ HL60 and THP‐1 cells were stably transfected with lentivirus wrapped sh‐circTASP1#1, sh‐circTASP1#2 or sh‐NC plasmids (RiboBio). The miR‐515‐5p mimic, miR‐NC mimic or anti‐miR‐515‐5p (GenePharma) were transfected into HL60 and THP‐1 cell by Lipofectamine 2000™ (Invitrogen). Overexpressing HMGA2 (HMGA2) and blank plasmids (NC; 5 µg/well of each plasmid) were synthetized by Invitrogen and were transfected into HL60 and THP‐1 cells via Lipofectamine 2000™.

### 2.3 RNA‐FISH assay

The location of circTASP1 in cells was determined by RNA‐FISH assay. Cy3‐labelled circTASP1 probes were purchased from RiboBio, and the RNA‐FISH assay was performed by fluorescent in situ hybridization kit (RiboBio). After incubated with prehybridization solution for 3 hours at 37℃, the slices were blocked with Cy3‐labelled circTASP1 probes at 55℃ overnight. After washed with PBS for 3 times, the slices were counterstained with DAPI for 90 seconds. Finally, the images were captured with a fluorescence microscope (Olympus).

### 2.4 Cell Counting Kit‐8 (CCK‐8) assay

The cell viability of HL60 and THP‐1 cells was detected by CCK‐8 assay using Cell Counting Kit‐8 (CCK‐8; Beyotime). After treatment, a total of 5 × 10^3^ cells were seeded in each 96‐well plate and incubated for 24 hours, 48 hours and 72 hours, respectively. Each well was added with 10 μL CCK8 reagent for 1‐hour incubation. Finally, the OD 450 nm value of each sample was examined by a microplate reader.

### 2.5 5‐Ethynyl‐20‐deoxyuridine (EdU) incorporation assay

The EdU detection was performed by Cell‐Light EdU DNA Cell proliferation kit (RiboBio). After incubated with 50 mmol/L EdU for 2 hours, the HL60 and THP‐1 cells were fixed in 4% paraformaldehyde and then stained with Apollo Dye Solution (cell nucleus was stained by DAPI). The EdU‐positive cells were photographed and counted using a fluorescence microscope.

### 2.6 Apoptosis and cell cycle analysis

After washed with PBS, the HL60 and THP‐1 cell apoptosis and cell cycle were detected by Annexin V‐FITC Apoptosis Detection Kit (Beyotime) and EZCellTM Cell Cycle Analysis Kit (BioVision), respectively. The cell apoptosis and cell cycle were examined using flow cytometer (Beckman).

### 2.7 Luciferase reporter assay

The wild‐type and mutational miR‐515‐5p binding site of circTASP1 or miR‐515‐5p binding site of HMGA2 was inserted into pGL3 vector (Promega). MiR‐515‐5p mimic or miR‐NC mimic was co‐transfected with circTASP1‐MUT, circTASP1‐WT, HMGA2‐MUT or HMGA2‐WT into HL60 and THP‐1 cells. The luciferase activity was detected by a Dual‐Luciferase Reporter Assay Kit (Promega).

### 2.8 RNA immunoprecipitation (RIP) assay

EZ‐magna RIP RNA‐binding protein immunoprecipitation kit (Millipore) was used for RIP detection to investigate the interaction between circTASP1 and mir‐515‐5p. After lysis of cells, the cells were incubated with Millipore antibody against human Ago2 coated on magnetic beads in RIP buffer solution. The input and IgG were considered as controls. The RNA was isolated and reverse transcribed into cDNA, and the circTASP1 and miR‐515‐5p levels were analysed by qRT‐PCR.

### 2.9 Biotinylated RNA pull‐down assay

Pierce RNA 3’‐End Desthiobiotinylation Kit (Thermo Fisher Scientific) was used to label purified RNAs with biotin in cell lysates, which were then named as biotin‐labelled miR‐515‐5p (miR‐515‐5p‐Bio), miR‐NC‐Bio and miR‐515‐5p‐Bio‐MUT. Each binding reaction was detected by magnetic beads and eluted RNA was detected by qRT‐PCR.

### 2.10 Quantitative real‐time PCR (qRT‐PCR) assay

Total RNA from peripheral blood and cells was isolated by TRIzol reagent (Invitrogen). The reverse transcription kit (Takara) was used to synthesize cDNA. The gene expression was examined by qRT‐PCR using SYBR‐Green kit (TaKaRa) and detected using StepOnePlus™ real‐time PCR system (Applied Biosystems). The GAPDH or U6 is considered as an internal control. Each sample is repeated at least 3 times.

### 2.11 Western blot

Proteins from cells were extracted and separated by SDS‐PAGE. After transferred onto PVDF membranes (Millipore), the proteins were blocked with 5% skim milk and then incubated with Bax antibody (dilution 1:800; Abcam), cleaved caspase 3 antibody (dilution 1:800; Abcam), Bcl‐2 antibody (dilution 1:800; Abcam), HMGA2 antibody (dilution 1:800; Abcam) or GAPDH antibody (dilution 1:2000; Abcam) at 4℃ overnight. After washed with TBST buffer, the membranes were incubated with HRP‐conjugated secondary antibodies (dilution 1:5000; Beyotime) and the bands were visualized using BeyoECL Moon (Beyotime).

### 2.12 Tumour xenografts

A total of 10 BALB/c‐Nude mice (Charles River, China) were randomly divided into two groups: sh‐NC group and sh‐circTASP1 group (5 mice for each group). A total of 5 × 10^6^ HL60 cells were stably transfected with lentivirus wrapped sh‐circTASP1 or sh‐NC plasmids and then subcutaneously injected into the right flank of each mouse. After 15 days, the tumours were measured every 5 days and then removed on 40th day.

### 2.13 Immunohistochemistry (IHC) staining

Ki67 and cleaved caspase 3 expressions were determined using IHC assay. The tumours were embedded with paraffin, cut into sections, dewaxed and hydrated. Antigens were retrieved by citrate buffer and blocked with 3% H_2_O_2_ at room temperature for 15 minutes. Goat serum was used to block nonspecific binding sites for 15 minutes. Slides were incubated with Ki67 (dilution 1:600; Abcam) or cleaved caspase 3 antibodies (dilution 1:600; Abcam) at 4℃ overnight followed by the secondary antibody for 20 minutes at room temperature and then incubated in streptavidin‐horseradish peroxidase (SA‐HRP) solution for 20 minutes. The slides were finally stained by diaminobenzidine (DAB) (ZSGB‐BIO) and haematoxylin.

### 2.14 Statistical analysis

SPSS software 20.0 was used for statistical analyses. The *t* test or one‐way analysis of variance was utilized to analyse the differences between groups. The correlations between circTASP1 and miR‐515‐5p expression, circTASP1 and HMGA2 expression, and miR‐515‐5p and HMGA2 expression in patients with AML were evaluated by Pearson correlation analysis. Kaplan‐Meier method with log‐rank test was used for survival analysis. The data (mean ± SD) were calculated, and *P* < .05 was considered significant.

## 3 RESULTS

### 3.1 CircTASP1 was up‐regulated in AML patients and cells

**FIGURE 1:**
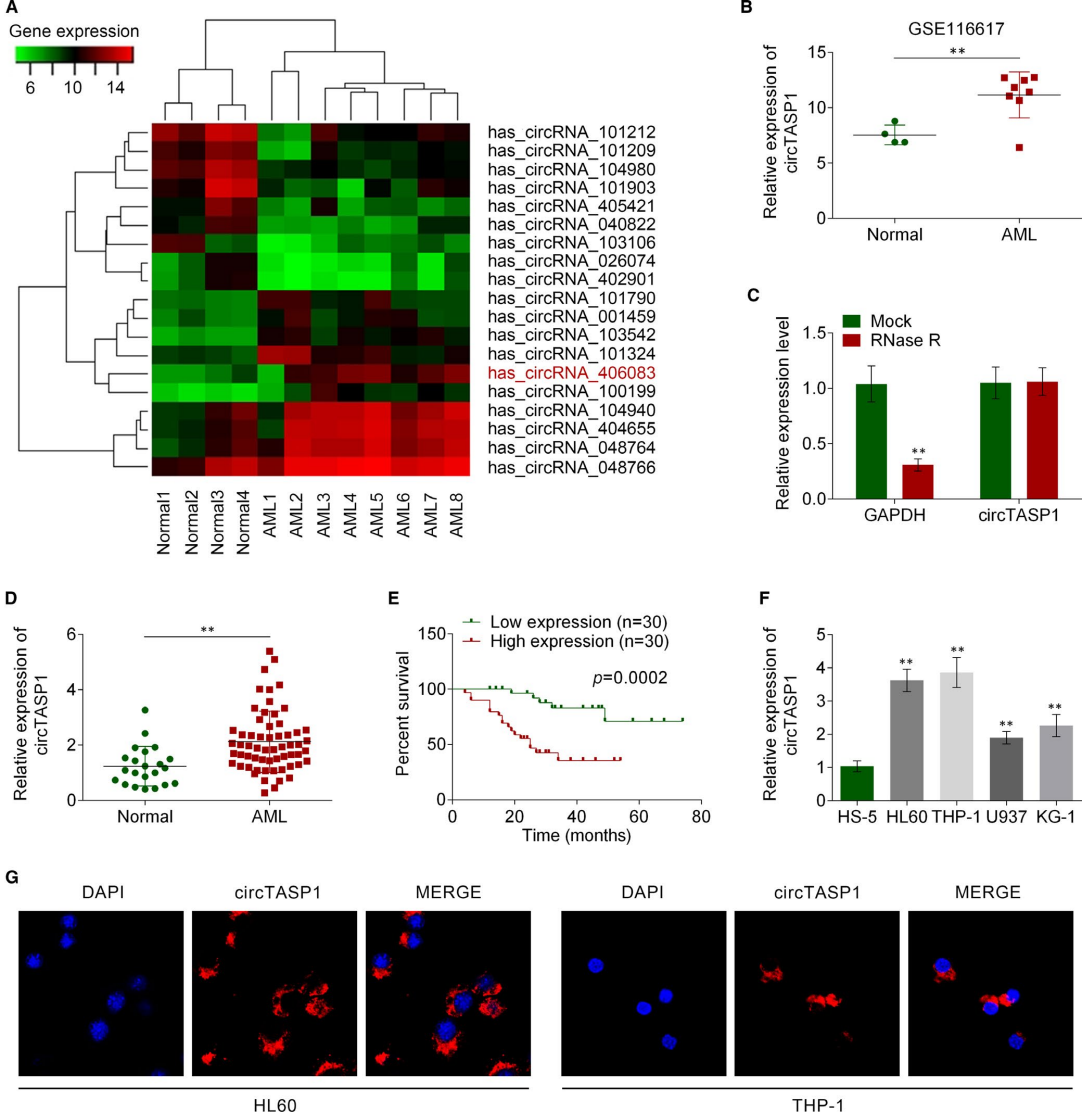
CircTASP1 was up‐regulated in AML patients and cells. A, Heat map of 19 expressed circRNAs AML patients and normal healthy individuals according to circRNA microarray data set (GSE116617). B, Expression of circTASP1 was up‐regulated in 8 patients with AML compared with 4 normal individuals, which was detected by qRT‐PCR assay. C, CircTASP1 expression could not be influenced by RNase R digestion. D, CircTASP1 expression in patients with AML was higher than that in normal individuals as detected by qRT‐PCR. E, Kaplan‐Meier curve of survival of patients with AML according to circTASP1 expression (mean value as cut‐off). F, CircTASP1 was up‐regulated in AML cell lines (HL60, THP‐1, U937 and KG‐1). G, RNA‐FISH was utilized to examine the localization of circTASP1 in HL60 and THP‐1 cells. *P < .05; **P < .01. Each experiment was repeated 3 times

Based on the circRNA microarray GSE116617, 19 remarkably differentially expressed circRNAs were screened based on the fold change value between 8 patients with AML and 4 normal healthy individuals via bioinformatics analyses. The data were visualized in a clustered heat map, among which hsa_circRNA_406083 (hsa_circ_0007340; chromosome 20, 13509092 to 13561628; Gene symbol: TASP1) was highly expressed in patients with AML compared with normal individuals (Figure 1A). The Sanger sequencing of the head‐to‐tail splicing in the RT‐PCR product of circTASP1 was performed in Figure S2. In order to validate the bioinformatics analyses, the expression of circTASP1 was determined by qRT‐PCR assay in 8 patients with AML and 4 normal individuals, and the results showed that circTASP1 was remarkably up‐regulated in patients with AML compared with normal individuals (Figure 1B). In addition, after the RNase R digestion, the expression of circTASP1 remained unchanged, indicating that circTASP1 was a circle RNA and could not be digested by RNase R (Figure 1C). Subsequently, we evaluated circTASP1 expression in peripheral blood from 60 patients with AML and 20 normal individuals. CircTASP1 expression was obviously higher in patients with AML (Figure 1D). In addition, the CD34^+^ cells were isolated from peripheral blood of 60 patients with AML and 20 normal individuals. As we expected, the cirTASP1 was up‐regulated in CD34^+^ cells of patients with AML compared with healthy individuals (Figure S4). According to the cut‐off value, we classified the whole‐cohort of patients with AML into two groups (high and low) in order to further analyse the clinical significance of circTASP1 expression in AML. High circTASP1 expression was found to be associated with CEBPα (*P* = .037) and haemoglobin (HGB) (*P* = .018, Table 1). Moreover, a multivariate analysis showed significant differences between circTASP1 and HGB (*P* = .002; Table 2). In addition, the Kaplan‐Meier analysis indicated that high circTASP1 levels led to poor survival of patients with AML (Figure 1E). Furthermore, circTASP1 was also significantly up‐regulated in AML cell lines (HL60, THP‐1, U937 and KG‐1) compared with normal cell lines (HS‐5) (Figure 1F). To confirm the location of circTASP1 in cells, the RNA‐FISH assay was performed, and the results indicated that circTASP1 was located in the cytoplasm of HL60 and THP‐1 cells (Figure 1G). Given that circTASP1 was up‐regulated in patients with AML and cells, we decided to focus on the function of circTASP1 in further experiments.

**TABLE 1:** Relationship between circTASP1 expression and clinical characteristics of patients with acute myeloid leukaemia

Characteristics	Number of patients	circTASP1 High expression	circTASP1 Low expression	*P* value
Age (mean)		49.3 ± 19	51.08 ± 20	.752
Sex				
Male	36	17	19	.598
Female	24	13	11	
WBC/ × 10^9^/L				
≥10	47	23	24	.754
<10	13	7	6	
PLT × 10^9^/L				
≥50	25	13	12	.793
<50	35	17	18	
HGB (g/L)				
≥90	35	22	13	.018^*^
<90	25	8	17	
CEBPα				
Positive	26	9	17	.037^*^
Negative	34	21	13	
NPM1				
Mutation	19	9	10	.781
Wild type	41	21	20	
FLT3‐ITD				
Positive	24	14	10	.292
Negative	36	16	20	
IDH1/2				
Positive	18	11	7	.260
Negative	42	19	23	
DNMT3A				
Positive	30	16	14	.606
Negative	30	14	16	
FAB subtypes				
M1	9	5	4	.661
M2	31	13	18	
M3	4	2	2	
M4	12	7	5	
M5	2	2	0	
M6	2	1	1	
Marrow blast				
≥0.6000	34	15	19	.297
<0.6000	26	15	11	
Karyotype				
Normal	43	21	22	.554
Complex	10	4	6	
inv(16)/CBFβ‐MYH11	1	1	0	
Others	6	4	2	

^*^ *P* < .05.

**TABLE 2:** Analysis of prognostic factors in acute myeloid leukaemia

Characteristics	Wald	HR (95% Cl)	*P* value
circTASP1 expression	18.778	20.778 (5.268 ~ 81.953)	.000^*^
WBC/ × 10^9^/L	1.393	1.004 (0.998 ~ 1.010)	.238
PLT × 10^9^/L	0.519	1.012 (0.979 ~ 1.047)	.471
HGB (g/L)	9.778	0.974 (0.959 ~ 0.990)	.002^*^
CEBPα	0.491	0.573 (0.121 ~ 2.721)	.483
NPM1	1.435	3.474 (0.453 ~ 26.662)	.231
FLT3‐ITD	0.116	1.341 (0.249 ~ 7.227)	.733
IDH1/2	0.433	1.796 (0.314 ~ 10.254)	.510
DNMT3A	0.444	1.657 (0.375 ~ 7.322)	.505
FAB subtypes	2.042	0.465 (0.163 ~ 1.329)	.153
Marrow blast	0.003	1.082 (0.059 ~ 20.011)	.958
Karyotype	2.394	0.505 (0.213 ~ 1.200)	.122

^*^ *P* < .05.

### 3.2 Knockdown of circTASP1 inhibited proliferation and induced apoptosis in AML cell lines

**FIGURE 2:**
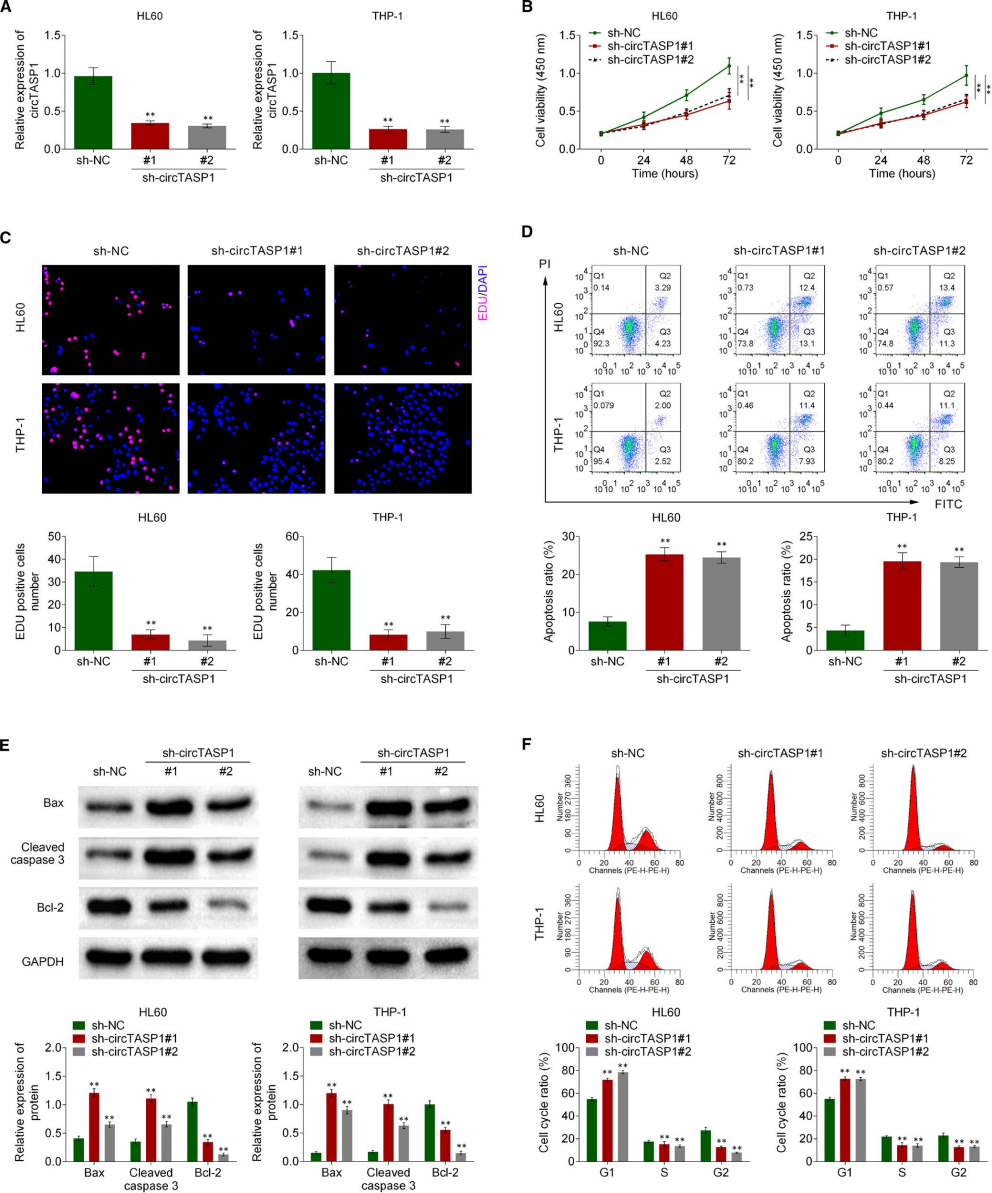
Knockdown of circTASP1 inhibited proliferation and induced apoptosis in AML cell lines. The HL60 and THP‐1 cells were transfected with sh‐circTASP1#1 or sh‐circTASP1#2. A, The expression of circTASP1 in HL60 and THP‐1 cells was examined by qRT‐PCR assay. B, The cell viability of HL60 and THP‐1 cells was evaluated by CCK‐8 assay. C, The HL60 and THP‐1 cell proliferation was detected by Edu staining assay. D, Cell apoptosis in HL60 and THP‐1 cells was determined using flow cytometry assay. E, The Bax, cleaved caspase 3 and Bcl‐2 expression in HL60 and THP‐1 cells were detected by Western blot. F, The cell cycle distributions in HL60 and THP‐1 cells were determined using flow cytometry assay. **P < .01. Each experiment was repeated 3 times

To verify the role of circTASP1 in AML, sh‐circTASP1#1 or sh‐circTASP1#2 was transfected into HL60 and THP‐1 cells. The results showed that sh‐circTASP1#1 or sh‐circTASP1#2 could significantly reduce the expression of circTASP1 but could not change the linear TASP1 mRNA levels in both HL60 and THP‐1 cells (Figure 2A and Figure S1). Through CCK‐8 assay, we found that knockdown of circTASP1 impaired the cell viability of HL60 and THP‐1 cells (Figure 2B). Additionally, Edu staining confirmed that knockdown of circTASP1 decreased the Edu‐positive cells, indicating that the cell proliferation was inhibited by knockdown of circTASP1 in HL60 and THP‐1 cells (Figure 2C). Flow cytometry analysis results suggested that knockdown of circTASP1 increased cell apoptosis rate in HL60 and THP‐1 cells (Figure 2D). Moreover, Western blot assay indicated that knockdown of circTASP1 induced Bax and cleaved caspase 3 expression but inhibited Bcl‐2 expression in HL60 and THP‐1 cells (Figure 2E). Aberrant cell cycle results in decreased proliferation and increased apoptosis. Cell cycle analysis suggested that knockdown of circTASP1 led to more cells arrested in G1 phase while less cells in S and G2 phase in HL60 and THP‐1 cells (Figure 2F). These findings indicated that knockdown of circTASP1 inhibited proliferation and induced apoptosis in HL60 and THP‐1 cells.

### 3.3 CircTASP1 functioned as a molecular sponge of miR‐515‐5p

**FIGURE 3:**
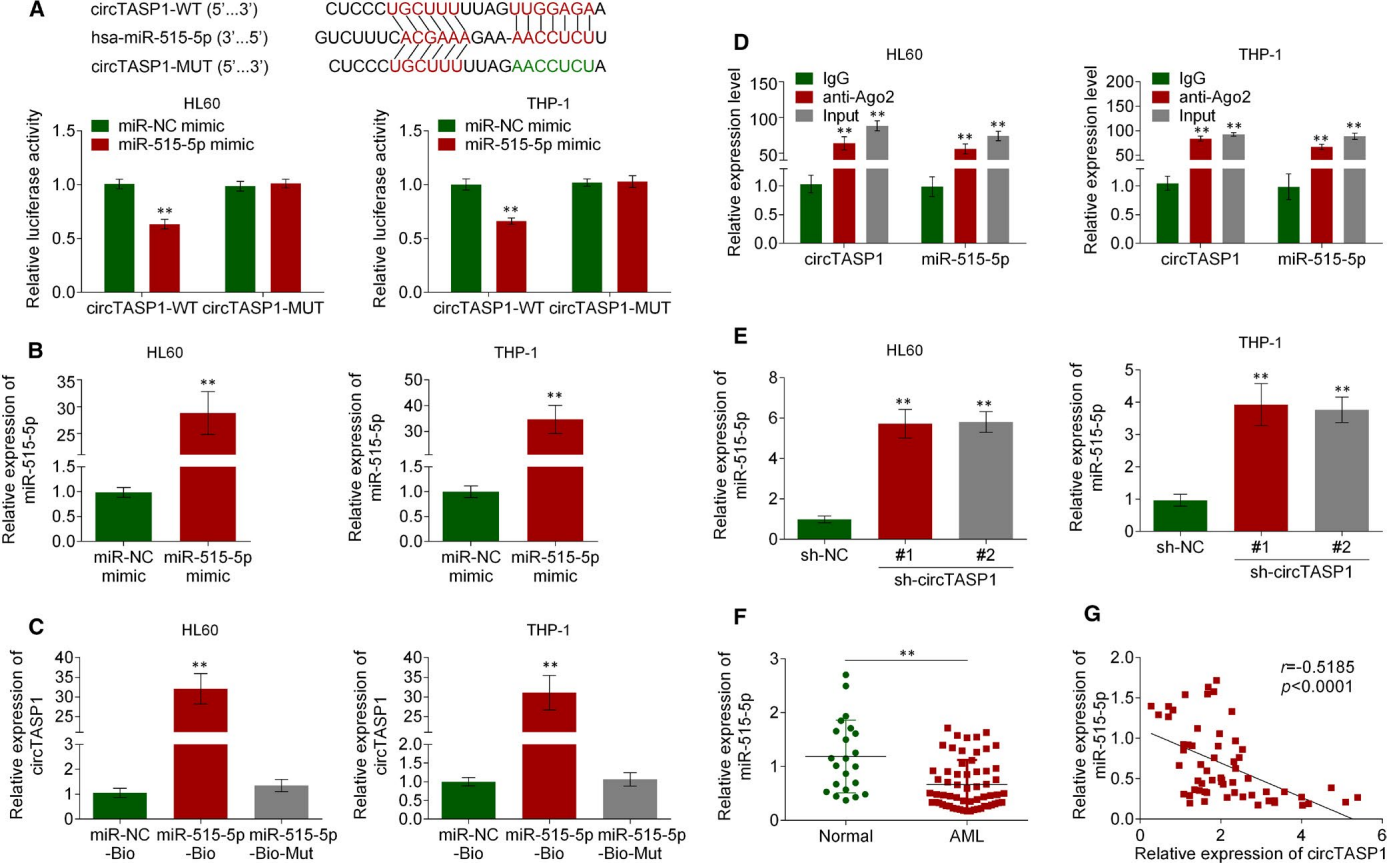
CircTASP1 functioned as a molecular sponge of miR‐515‐5p. A, The binding site of miR‐515‐5p and circTASP1 was predicted by circinteractome, and the interaction of miR‐515‐5p and circTASP1 was examined by luciferase activity assay. B, QRT‐PCR assay suggested that miR‐515‐5p mimic significantly increased miR‐515‐5p expression in HL60 and THP‐1 cells. C, RNA pull‐down assay was performed with miR‐515‐5p‐biotin, miR‐515‐5p‐biotin‐MUT or miR‐NC‐biotin using HL60 and THP‐1 cell extracts. RNA levels of circTASP1 in immunoprecipitates were detected by qRT‐PCR. D, RIP assay indicated that circTASP1 and miR‐515‐5p were specifically bound by AGO2. E, QRT‐PCR assay confirmed that knockdown of circTASP1 promoted miR‐515‐5p expression in HL60 and THP‐1 cells. F, The qTR‐PCR analysis of the relative expression of miR‐515‐5p in patients with AML and normal individuals. G, Pearson's correlation coefficients were used to evaluate the correlation between circTASP1 and miR‐515‐5p in patients with AML. **P < .01. Each experiment was repeated 3 times

CircRNAs may function as sponges for miRNAs and interact with RISC complex.^13^ To verify the mechanism of circTASP1, the potential target of circTASP1 was predicted by circinteractome (https://circinteractome.nia.nih.gov/). Fortunately, miR‐515‐5p was identified as the potential target of circTASP1. Subsequently, luciferase activity assay confirmed that miR‐515‐5p mimic inhibited the activity of circTASP1‐WT reporter in HL60 and THP‐1 cells (Figure 3A). Further, miR‐515‐5p mimic significantly promoted miR‐515‐5p expression in HL60 and THP‐1 cells (Figure 3B). To examine the interaction between miR‐515‐5p and circTASP1, the 3’‐terminal‐biotinylated‐miR‐515‐5p probe or 3’‐terminal‐biotinylated‐miR‐515‐5p‐MUT probe was designed. The probe was validated to pull down circTASP1 in HL60 and THP‐1 cells (Figure 3C). AGO2 is a unique protein of RNA‐induced silencing complex (RISC) with endonuclease activity and recognizes specific targets through base‐pairing of small interfering RNA or microRNA (miRNA), resulting in degradation of mRNA.^14^ To validate this interaction, we performed RIP of AGO2 in HL60 and THP‐1 cells, and the results showed that both circTASP1 and miR‐515‐5p were specifically bound by AGO2 (Figure 3D). As shown in Figure 3E, miR‐515‐5p expression was promoted by the knockdown of circTASP1 in HL60 and THP‐1 cells. In addition, miR‐515‐5p was down‐regulated in peripheral blood from 60 patients with AML compared with 20 normal individuals (Figure 3F). More importantly, miR‐515‐5p expression was negatively correlated with circTASP1 in peripheral blood from 60 patients with AML (Figure 3G). Collectively, these data indicated that circTASP1 functioned as a molecular sponge of miR‐515‐5p.

### 3.4 Overexpressed miR‐515‐5p inhibited cell proliferation and promoted cell apoptosis

**FIGURE 4:**
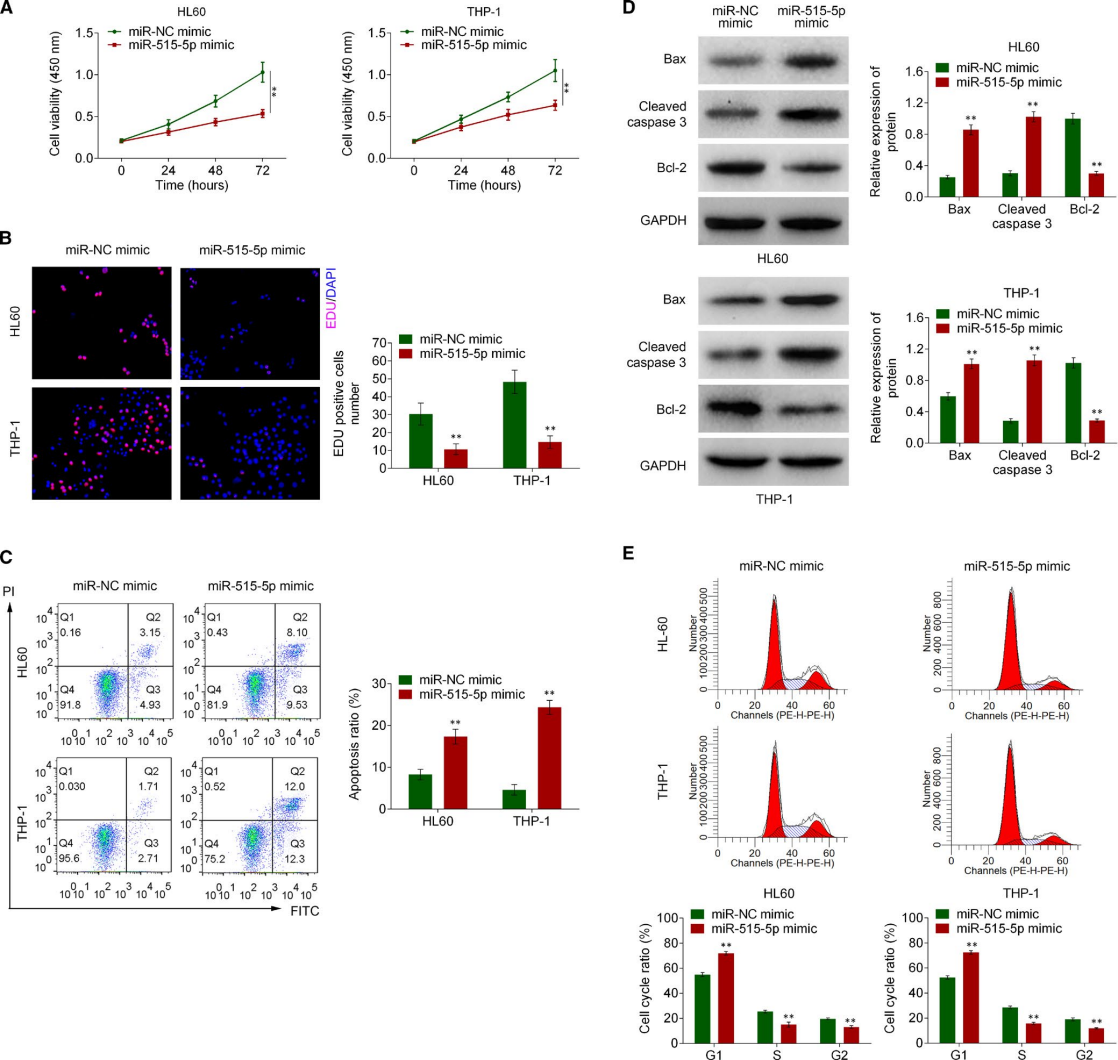
Overexpressed miR‐515‐5p inhibited cell proliferation and promoted cell apoptosis. The HL60 and THP‐1 cells were transfected with miR‐515‐5p mimic or miR‐NC mimic. A, The cell viability of HL60 and THP‐1 cells was evaluated by CCK‐8 assay. B, The HL60 and THP‐1 cell proliferation was detected by Edu staining assay. C, Cell apoptosis in HL60 and THP‐1 cells was determined using flow cytometry assay. D, The Bax, cleaved caspase 3 and Bcl‐2 expression in HL60 and THP‐1 cells were detected by Western blot. E, The cell cycle distributions in HL60 and THP‐1 cells were determined using flow cytometry assay. **P < .01. Each experiment was repeated 3 times

To identify the role of miR‐515‐5p in AML cells, miR‐515‐5p mimic was transfected into HL60 and THP‐1 cells. CCK‐8 assay confirmed that miR‐515‐5p mimic decreased cell viability of HL60 and THP‐1 cells (Figure 4A). Then, the Edu staining assay proved that miR‐515‐5p mimic reduced the Edu‐positive HL60 and THP‐1 cells, indicating that the cell proliferation was inhibited by overexpression of miR‐515‐5p in HL60 and THP‐1 cells (Figure 4B). Flow cytometry analysis suggested that miR‐515‐5p mimic promoted cell apoptosis rate in HL60 and THP‐1 cells (Figure 4C). Moreover, as shown in Figure 4D, miR‐515‐5p mimic induced Bax and cleaved caspase 3 expression while inhibited Bcl‐2 expression in HL60 and THP‐1 cells. Subsequently, cell cycle assay suggested that miR‐515‐5p mimic arrested more HL60 and THP‐1 cells in G1 phase (Figure 4E). These results suggested that miR‐515‐5p mimic suppressed proliferation and promoted apoptosis in HL60 and THP‐1 cells.

### 3.5 HMGA2 was a direct target of miR‐515‐5p

**FIGURE 5:**
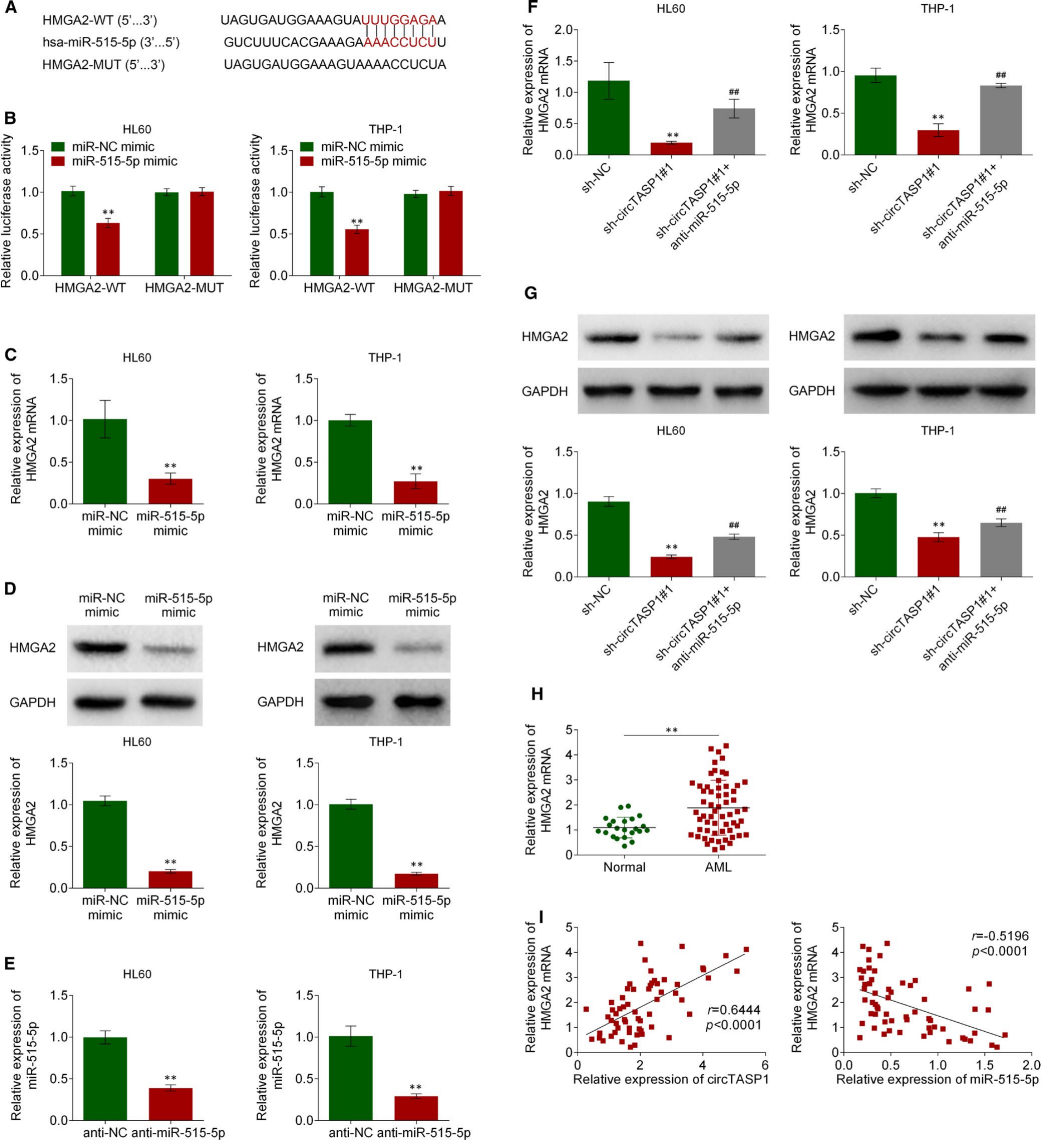
HMGA2 was a direct target of miR‐515‐5p. A, The binding of miR‐515‐5p with HMGA2 gene 3’‐UTR predicted by TargetScan. B, The direct binding between miR‐515‐5p and HMGA2 gene promoter was performed by dual‐luciferase reporter assay. C, HMGA2 mRNA levels in HL60 and THP‐1 cells transfected with miR‐515‐5p mimics. D, HMGA2 protein levels in HL60 and THP‐1 cells transfected with miR‐515‐5p mimics. E, The anti‐miR‐515‐5p decreased the miR‐515‐5p levels in HL60 and THP‐1 cells. F, QRT‐PCR assay confirmed that knockdown of circTASP1 reduced HMGA2 expression, which was reversed by anti‐miR‐515‐5p. G, Western blot confirmed that knockdown of circTASP1 reduced HMGA2 expression, which was reversed by anti‐miR‐515‐5p. H, QRT‐PCR assay proved that HMGA2 expression in patients with AML was higher than that in normal individuals. I, Negative correlation between miR‐515‐5p and HMGA2 expression in patients with AML. Positive correlation between circTASP1 and HMGA2 expression in patients with AML. **P < .01, ##*P* < .01. Each experiment was repeated 3 times

To clarify the molecular mechanism downstream of miR‐515‐5p during the development of AML, the TargetScan (www.targetscan.org) was used to predict the direct target of miR‐515‐5p, and the results showed that 3’‐UTR of HMGA2 gene bound with miR‐515‐5p (Figure 5A). Further, the luciferase assay indicated that miR‐515‐5p mimic significantly reduced luciferase activity of HL60 and THP‐1 cells expressing HMGA2‐WT, compared with miR‐NC mimic (Figure 5B). Moreover, HMGA2 expression was significantly repressed by miR‐515‐5p mimic in HL60 and THP‐1 cells (Figure 5C). Consistent with qRT‐PCR assay, HMGA2 protein level was markedly suppressed by transfection with miR‐515‐5p mimic in HL60 and THP‐1 cells (Figure 5D). Further, the anti‐miR‐515‐5p was transfected into HL60 and THP‐1 cells to reduce the miR‐515‐5p expression (Figure 5E). The results showed that knockdown of circTASP1 decreased the HMGA2 mRNA level, whereas this change was reversed by anti‐miR‐515‐5p (Figure 5F). Similarly, knockdown of circTASP1 reduced the HMGA2 protein level, while this change was reversed by anti‐miR‐515‐5p (Figure 5G). Additionally, the miR‐515‐5p expression was up‐regulated in peripheral blood from 60 patients with AML compared with 20 normal individuals (Figure 5H). Besides, the expression of HMGA2 was positively correlated with circTASP1 and negatively correlated with miR‐515‐5p in peripheral blood from 60 patients with AML (Figure 5I). Therefore, HMGA2 was a direct target of miR‐515‐5p in AML.

### 3.6 Knockdown of circTASP1 inhibited proliferation and induced apoptosis by modulating miR‐515‐5p/HMGA2 pathway

**FIGURE 6:**
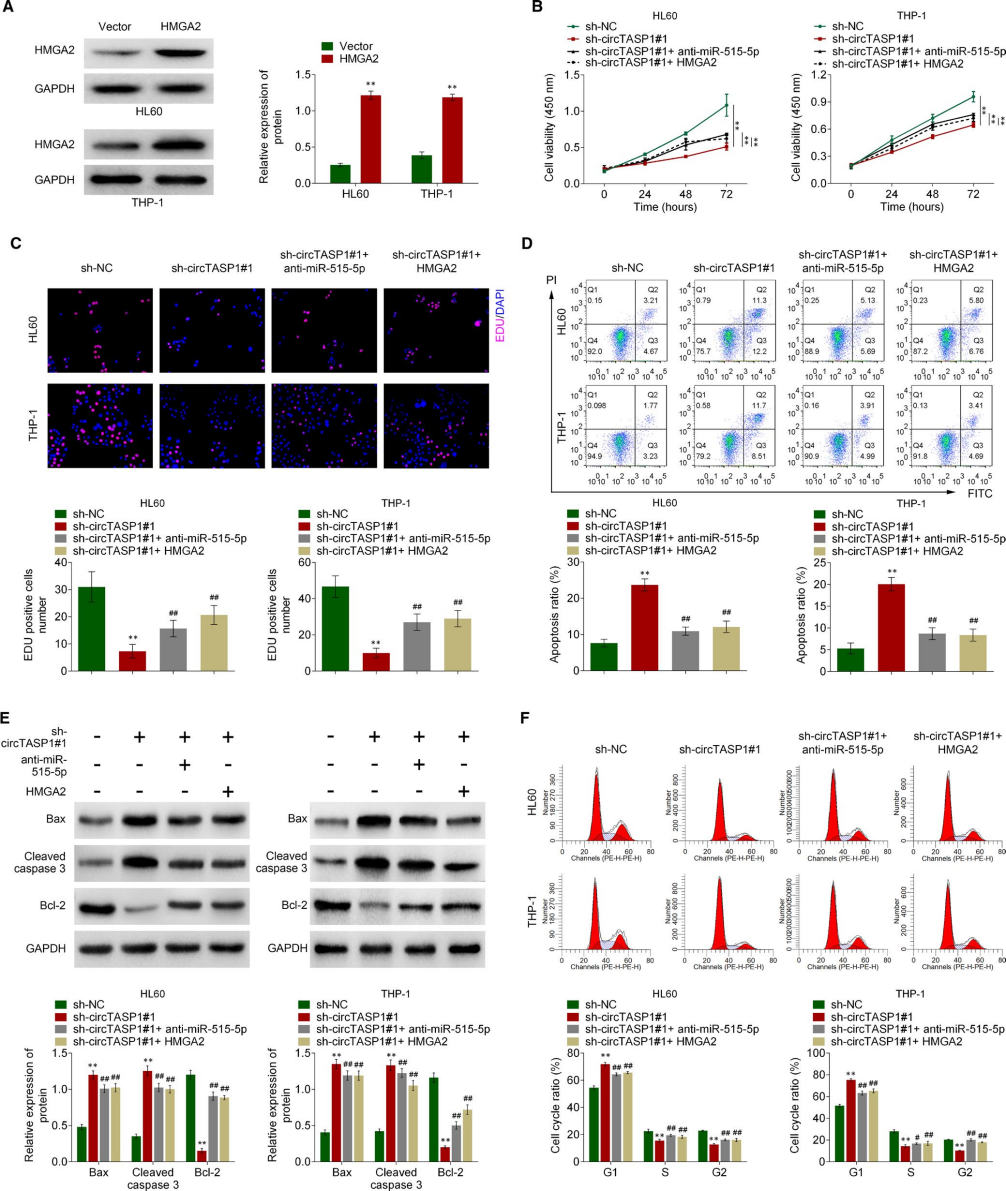
Knockdown of circTASP1 inhibited proliferation and induced apoptosis by modulating miR‐515‐5p/HMGA2 pathway. A, The transfection efficiency of HMGA2 was validated by Western blot. The CCK‐8 assay (B), Edu straining assay (C) and flow cytometry assay (D) indicated that knockdown circTASP1 inhibited cell viability and proliferation, while promoted apoptosis in HL60 and THP‐1 cells, and when co‐transfected with anti‐miR‐515‐5p or HMGA2, these effects were reversed. E, The Bax, cleaved caspase 3 and Bcl‐2 expression were determined by Western blot. F, The cell cycle distributions in HL60 and THP‐1 cells were determined using flow cytometry assay. **P < .01, #*P* < .05, ##*P* < .01. Each experiment was repeated 3 times

To further clarify whether circTASP1 exerts its effects by modulating miR‐515‐5p/HMGA2 pathway, the rescue assays were performed by transfection with anti‐miR‐515‐5p or HMGA2 into HL60 and THP‐1 cells. First, the transfection efficiency was validated by analysing HMGA2 protein levels (Figure 6A). As shown in Figure 6B, circTASP1 knockdown inhibited proliferation and induced apoptosis of HL60 and THP‐1 cells. However, this effect was partially attenuated by anti‐miR‐515‐5p or HMGA2 (Figure 6B‐D). Besides, circTASP1 knockdown enhanced Bax and cleaved caspase 3 expression, while inhibiting Bcl‐2 expression in HL60 and THP‐1 cells. Nevertheless, these changes were partially rescued by anti‐miR‐515‐5p or HMGA2 (Figure 6E). More importantly, knockdown of circTASP1 led to more cells arrested in G1 phase in HL60 and THP‐1 cells, whereas anti‐miR‐515‐5p or HMGA2 alleviated this effect (Figure 6F). These data concluded that circTASP1 repressed proliferation and promoted apoptosis by modulating miR‐515‐5p/HMGA2 pathway.

### 3.7 Knockdown of CircTASP1 suppressed AML progression in vivo

**FIGURE 7:**
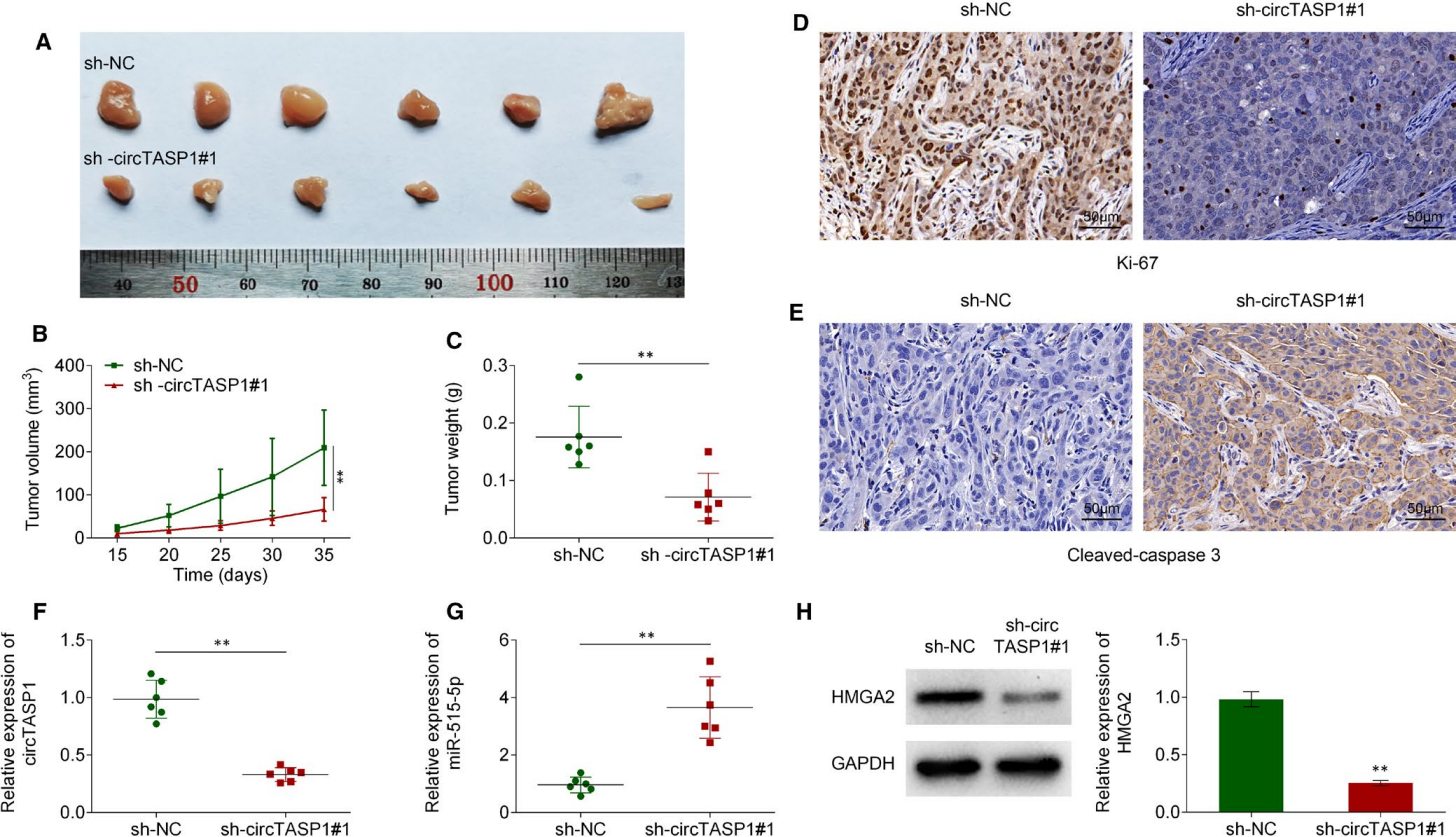
CircTASP1 knockdown suppressed AML progression in vivo. A, CircTASP1 knockdown significantly inhibited the tumour growth in *vivo*. Tumour volume (B) and tumour weight (C) of nude mice were analysed, n = 5. The expression of Ki67 (D) and cleaved caspase 3 (E) was determined by Immunohistochemistry. The expression of circTASP1 (F) and miR‐515‐5p (G) was determined using qRT‐PCR assay. H, The expression of HMGA2 was evaluated by Western blot. **P < .01

The role of circTASP1 in AML pathogenesis was determined by in vivo model. After transfected with sh‐circTASP1 or sh‐NC, the HL60 cells were subcutaneously injected into mice. As illustrated in Figure 7A‐C, knockdown of circTASP1 significantly reduced tumour volume and tumour weight. In addition, immunohistochemistry staining indicated that sh‐circTASP1 significantly reduced Ki67 expression while enhancing cleaved caspase 3 expression, indicating that knockdown of CircTASP1 suppressed cell apoptosis in vivo (Figure 7D, E). Moreover, circTASP1 was observed to be down‐regulated in tumours, accompanied by elevated miR‐515‐5p expression and decreased HMGA2 (Figure 7F‐H). The tumorigenicity of HL60 cells was significantly reduced by knocking down of circTASP1 gene, which further suggested that the pro‐tumorigenic role of circTASP1 was mediated by miR‐515‐5p/HMGA2 pathway in the pathogenesis of AML.

## 4 DISCUSSION

Multidisciplinary research on circRNA is increasing, indicating that circRNA can be used as regulators and valuable diagnostic markers for disease.^6^ In the present study, circRNA expression profiles were screened with circRNA microarray, and the results showed that circTASP1 was significantly up‐regulated in patients with AML. Moreover, silencing of circTASP1 inhibited AML cells growth and proliferation by sponging miR‐515‐5p in HL60 and THP‐1 cells. Given that HMGA2 was a direct target of miR‐51‐5p, silencing of circTASP1 inhibited proliferation and induced apoptosis through modulating miR‐515‐5p/HMGA2 axis in AML (Figure S3).

Circular TASP1 (circTASP1) is encoded by TASP1 gene.^15^ Protease Taspase 1 (threonine aspartase 1; TASP1) is first identified as an endopeptidase, and its modulation is responsible for the cleavage of mixed lineage leukaemia 1 (MLL1) protein to appropriately regulate HOX expression.^16^ Aberrant expression of TASP1 has been found in leukaemia and alters cell cycle progression, cell proliferation and apoptosis.^17^ AML is a malignant clonal disease with multiple prognostic outcome: 5‐year overall survival is less than half, and approximately 20 per cent of elderly patients survive within 2 years after diagnosis.^18^ CircTASP1 is one of the products of back‐spliced RNAs of TASP1.^19^ Considering the vital role of TASP1 in leukaemia, the potential function of circTASP1 in the prognosis of AML needs to be investigated. A recent study has demonstrated that circtasp1 is up‐regulated in T‐cell acute lymphoblastic leukaemia (T‐ALL).^11^ Similarly, we found that circTASP1 expression was significantly up‐regulated in patients with AML and high circTASP1 levels predicted poor survival of patients with AML, indicating that circTASP1 might be a promising diagnostic and prognostic biomarker for patients with AML. In the present study, knockdown of circTASP1 inhibited proliferation and induced apoptosis in AML cell lines, which was consistent with the function of TASP1. Our results for the first time suggested that circTASP1 positively regulates proliferation and apoptosis.

Previous studies have shown that the function of circRNAs is mainly to release miRNAs‐mediated inhibition of corresponding genes by targeting miRNAs.^20^ In AML, circ‐0004136 promotes cell proliferation via sponging miR‐142.^21^ Circular RNA‐100290 promotes cell proliferation and inhibits apoptosis through sponging miR‐203 in AML.^22^ Circ_0009910 suppresses cell proliferation and induces apoptosis through sponging miR‐20a‐5p in AML.^10^ These studies prove that circRNAs can sponge miRNAs and regulate cell proliferation and apoptosis, and ultimately participate in the occurrence of AML. Mechanistically, knockdown of circTASP1 inhibited proliferation and induced apoptosis via sponging miR‐515‐5p in AML cells. MiR‐515‐5p belongs to miR‐515 family and acts as a suppressor in breast cancer and lung cancer.^23^ In breast cancer and bladder cancer, miR‐515‐5p is involved in the ceRNA mechanism with LINC00673 and LncRNA SNHG3, respectively.^24^, ^25^ As the role of miR‐515‐5p in AML has never been illustrated, the data in this study proved that miR‐515‐5p was down‐regulated in AML cells and sponged by circTASP1.

HMGA2 is a member of the high mobility group superfamily and is widely considered as a novel oncogene.^26^ Accumulating studies have confirmed that HMGA2 is predominantly amplified and expressed in AML cells.^27^ In addition, highly expressed HMGA2 independently predicted the adverse clinical outcome for AML.^28^ Shuo Y et al have demonstrated that HMGA2 regulates AML progression via activating Wnt/β‐catenin signalling.^29^ In the current study, HMGA2 was identified as a direct target of miR‐515‐5p. Our study clarified that knockdown of circTASP1 sponged less miR‐515‐5p and reduced HMGA2 expression, thereby inhibiting proliferation and promoting apoptosis. Then, it may inactivate Wnt/β‐catenin signalling and lead to poor clinical outcome in AML. AML accounts for about 20% of childhood leukaemia.^30^ Based on our in vivo results, knockdown of circTASP1 in mice could significantly reduce tumour growth, indicating that circTASP1 can be considered as a potential therapeutic target for children's AML and save more lives. Although our study highlights the mechanism by which circTASP1 plays an important role in AML, there are still more possibilities to be revealed. It is commonly known that one circTASP1 may act as a sponge for multiple miRNAs thus exerting different functions in AML. Whether circTASP1 can be used as ceRNA for other miRNAs remains to be further studied.

In conclusion, our results showed that circTASP1 is up‐regulated in AML tissues and cells, and acts as a sponge for miR‐515‐5p to regulate HMGA2, thereby promoting proliferation and inhibiting apoptosis during AML progression. More importantly, circTASP1 can serve as a potential target for AML treatment.

## CONFLICT OF INTEREST

The authors state that there are no conflicts of interest to disclose.

## AUTHOR CONTRIBUTIONS

**Yuanyuan Lin:** Conceptualization (equal). **Yan Huang:** Data curation (equal). **Changda Liang:** Data curation (equal). **Shupei Xie:** Data curation (equal). **An Xie:** Conceptualization (equal).

## ETHICAL APPROVAL

All procedures in studies involving human participants were performed in accordance with the standards upheld by the Ethics Committee of Jiangxi Provincial Children's Hospital and with those of the 1964 Helsinki Declaration and its later amendments for ethical research involving human participants. All animal experiments were approved by the Medical Ethics Committee of The First Affiliated Hospital of Nanchang University for the use of animals and conducted in accordance with the National Institutes of Health Laboratory Animal Care and Use Guidelines.

## STATEMENT OF HUMAN AND ANIMAL RIGHTS

All procedures in studies involving human participants were performed in accordance with the standards upheld by the Ethics Committee of Jiangxi Provincial Children's Hospital and with those of the 1964 Helsinki Declaration and its later amendments for ethical research involving human participants. All animal experiments were approved by the Medical Ethics Committee of The First Affiliated Hospital of Nanchang University for the use of animals and conducted in accordance with the National Institutes of Health Laboratory Animal Care and Use Guidelines.

## STATEMENT OF INFORMED CONSENT

Written informed consent was obtained from a legally authorized representatives for anonymized patient information to be published in this article.

## Supporting information

Fig S1

Fig S2

Fig S3

Fig S4

## Data Availability

All data generated or analysed during this study are included in this published article.
