# Supplementary figures and images for: Silencing of circTASP1 inhibits proliferation and induces apoptosis of acute myeloid leukaemia cells through modulating miR‐515‐5p/HMGA2 axis
Source: J Cell Mol Med. 2021 Jul 1;25(15):7367–80. doi: 10.1111/jcmm.16765 (PMC8335685; doi:10.1111/jcmm.16765)

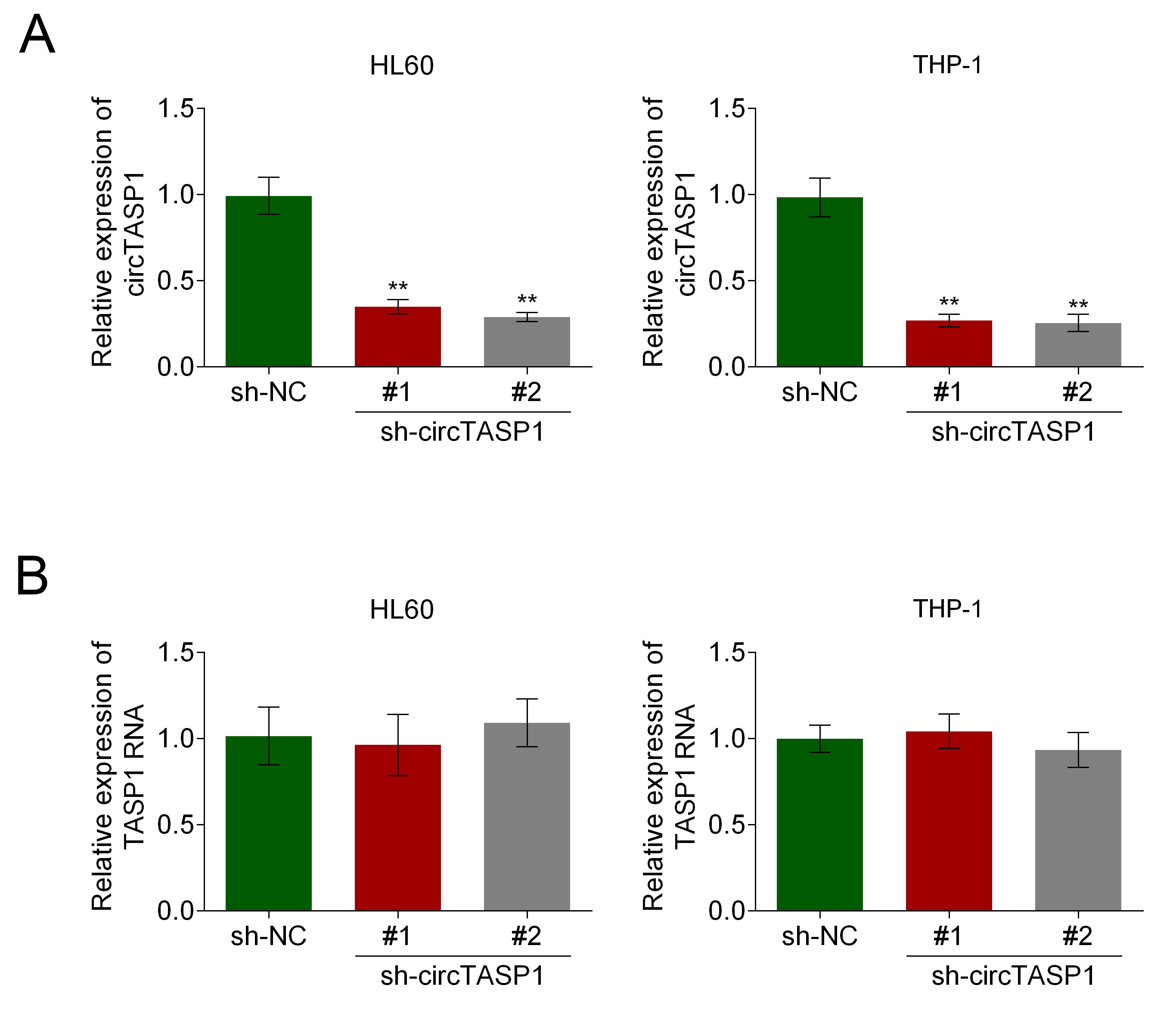

Supplement: Supplementary file 1 — Fig S1 [file JCMM-25-7367-s001.jpg]

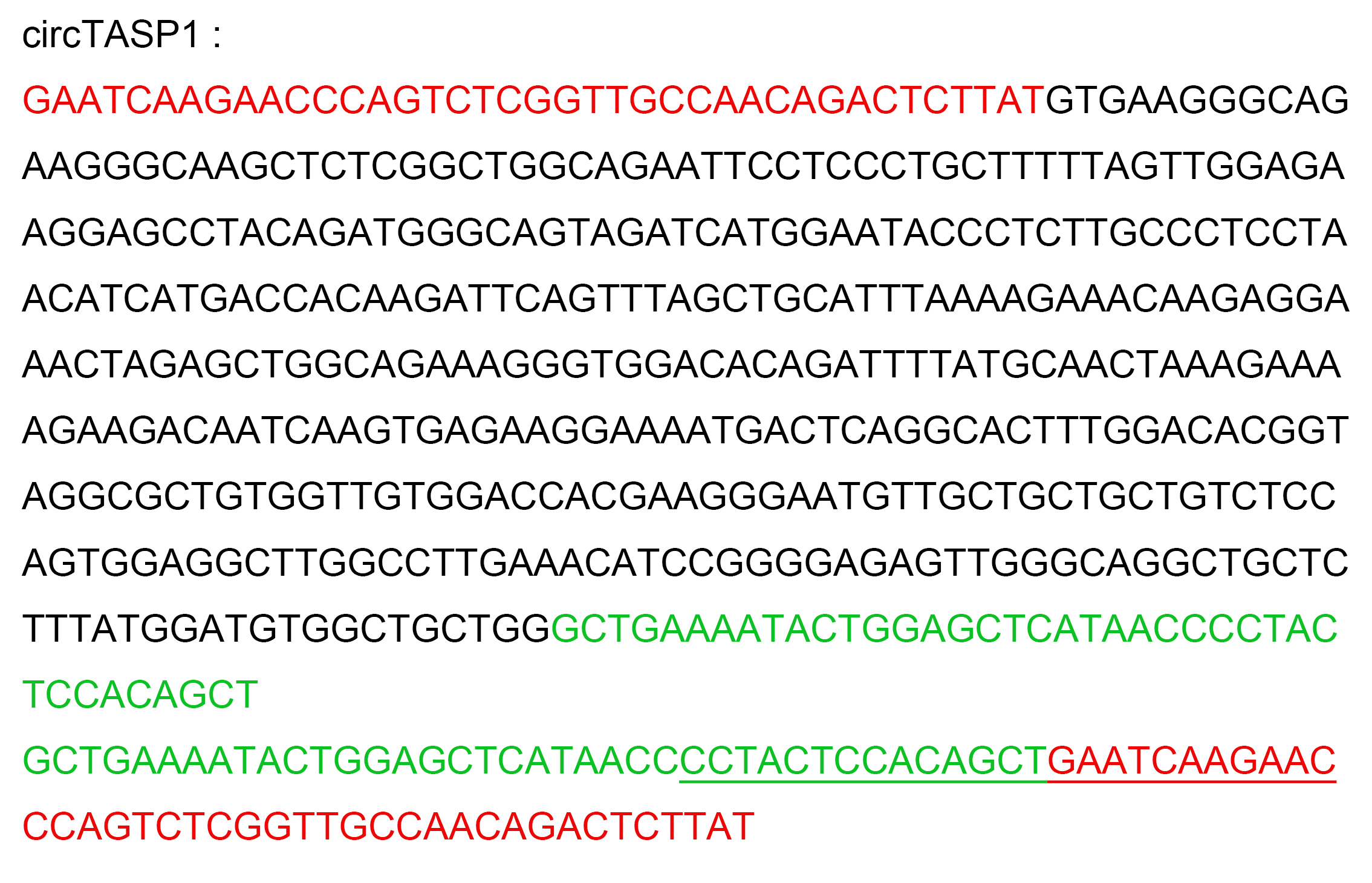

Supplement: Supplementary file 2 — Fig S2 [file JCMM-25-7367-s004.jpg]

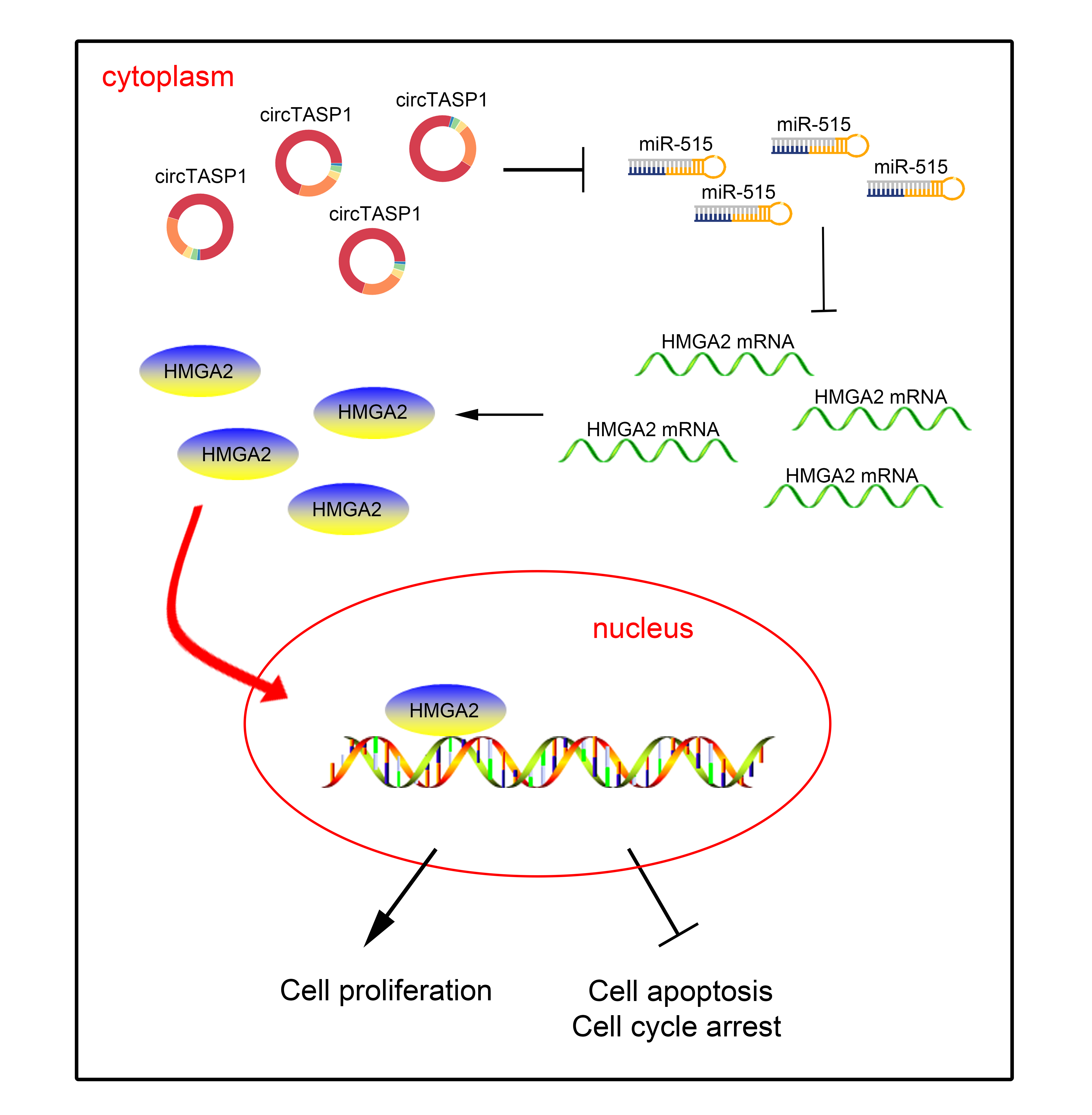

Supplement: Supplementary file 3 — Fig S3 [file JCMM-25-7367-s003.jpg]

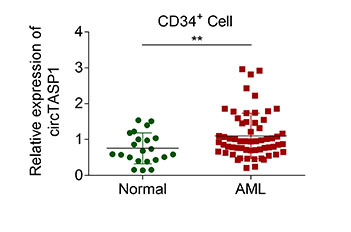

Supplement: Supplementary file 4 — Fig S4 [file JCMM-25-7367-s002.jpg]
